# Multimorbidity patterns, polypharmacy and their association with liver and kidney abnormalities in people over 65 years of age: a longitudinal study

**DOI:** 10.1186/s12877-020-01580-1

**Published:** 2020-06-12

**Authors:** Noemí Villén, Marina Guisado-Clavero, Sergio Fernández-Bertolín, Amelia Troncoso-Mariño, Quintí Foguet-Boreu, Ester Amado, Mariona Pons-Vigués, Albert Roso-Llorach, Concepción Violán

**Affiliations:** 1 Àrea del Medicament i Servei de Farmàcia, Gerencia Territorial de Barcelona, Institut Català de la Salut, Barcelona, Spain; 2 Fundació Institut Universitari per a la recerca a l’Atenció Primària de Salut Jordi Gol i Gurina (IDIAPJGol), Barcelona, Spain; 3 Universitat Autònoma de Barcelona, Cerdanyola del Vallès, Bellaterra, Spain; 4 Department of Psychiatry, Vic University Hospital, Vic, Spain; 5 Àrea de Serveis Assistencials. Servei Català de la Salut, Barcelona, Spain

**Keywords:** Multimorbidity, Epidemiology, Polypharmacy, Primary health care, Ageing, Longitudinal study

## Abstract

**Background:**

The implementation of individual clinical practice guidelines in patients with multimorbidity often results in polypharmacy. Our aim was to analyse medication use according to longitudinal multimorbidity patterns (MP) and determine during a 5-year period (2012–16) which MP are associated with abnormal liver and kidney function in primary care patients over 65 years of age living in Catalonia.

**Methods:**

*Design*: Longitudinal study (years 2012 to 2016) based on the electronic health records contained in Information System for Research in Primary Care database of the Catalan Institute of Health (SIDIAP). *Variables*: age, sex, MP, medication and polypharmacy (drug exposure obtained from the Pharmacy Invoice Registry). Medicines were classified in accordance with the Anatomical Therapeutic Chemical Classification System (ATC). Glomerular filtration rate was used to determine abnormal kidney function, and serum levels of alkaline phosphatase, alanine transaminase and gamma-glutamyl transpeptidase were used to diagnose abnormal liver function. *Statistics*: For medication use in MP, we calculated annual mean packages of each drug in each MP, and observed/expected ratios were obtained by dividing mean packages in the cluster by mean packages of the same drug in the overall population. Logistic regression models were fitted to estimate the association between MP at baseline and abnormal kidney and liver function tests during follow up.

**Results:**

Nine hundred sixteen thousand six hundred nineteen patients were included, and 743,827 completed the follow up. We identified one polypharmacy profile per MP, and concluded that the most prescribed drugs in each pattern corresponded to the diseases overrepresented in that specific MP. The median of drugs ranged from 3 (Cluster 1 - Non-Specific) to 8 (Cluster 10 - Multisystem Pattern). Abnormal kidney function was most commonly observed in the Cluster 4 - Cardio-Circulatory and Renal (Odds Ratio [OR] 2.19; Confidence interval [CI] 95% 2.15–2.23) and Cluster 3 - Minority Metabolic Autoimmune-Inflammatory (OR 2.16; CI 95% 2.12–2.20) MP. A higher risk of abnormal liver function was observed in the Cluster 8 - Digestive (OR 3.39; CI 95% 3.30–3.49), and Cluster 4 - Cardio-Circulatory and Renal (OR 1.96; CI 95% 1.91–2.02) MP.

**Conclusions:**

A higher risk of abnormal kidney and liver function was observed in specific MP. The long-term characterisation of MP and polypharmacy illustrates the burden of chronic multimorbidity and polypharmacy in the elderly population.

## Background

Multimorbidity generally refers to the co-occurrence of multiple chronic medical conditions in a single individual [1]. Globally, multimorbidity is growing into a major concern due to the longer lifespan of the population, the complexity of their health status and its relation to greater use of healthcare services [2, 3]. Additionally, living with multiple chronic diseases is commonly associated with the use of numerous drugs, function decline, lower of quality of life and increased mortality [4, 5].

Generally, prescription in patients with multimorbidity is based on the recommendations of individual disease-specific clinical practice guidelines. This frequently results in polypharmacy [6], commonly defined as the concomitant consumption of 5 or more drugs [7]. Polypharmacy is closely related to drug-drug and drug-disease interactions, therapeutic redundancies and adverse drug reactions that can cause hospital admission, worsen chronic conditions and increase morbidity and mortality, especially in the elderly and frail population [8–10]. Moreover, many drugs used to treat chronic diseases have a modest benefit at most and an increased risk of adverse reactions in this population.

The dynamic changes in cellular biological processes associated with aging [11] influence the pharmacokinetics and pharmacodynamics of drug metabolization [12]. Importantly, the mitochondrial function decline linked to the aging process increases the vulnerability of organs such as heart, lungs, liver and kidney to ischemia/reperfusion injury [13]. Consequently, understanding the link between polypharmacy and specific multimorbidity patterns (MP) and their interrelation with liver and kidney function decline is crucial for the design of strategies that prevent adverse effects of medication among the elderly. Additionally, there is a lack of longitudinal information about the effects of polypharmacy on adverse drug reactions and dynamic cellular changes associated with aging.

The pharmacological management of multimorbidity represents a challenge for health professionals, especially in elderly or frail patients, since it requires a person-centred approach as opposed to treating multiple diseases as independent entities [14]. Few publications address medication use according to MP, and to our knowledge this is the first study which includes long-term data on this issue [15–19].

In another article, we described MP in people over 65 years of age and followed their trajectories during 5 years. We used soft clustering techniques, which enabled us to study MP considering as the unit of analysis the patient plus all their diagnosed chronic diseases. We identified multimorbidity trajectories which were mostly stable [20]. In addition, we observed that nine over ten patterns involved a large number of chronic diseases and drugs. However, that study did not analyse the characteristics and use of medicines associated with MP over time, and their association with liver and kidney function abnormalities.

The main aim of this study was to analyse medication use according to longitudinal MP and characterize them annually in primary care patients over 65 years of age during the 2012–16 period in Catalonia. The secondary aim was to determine which patterns are more likely to present an abnormal liver and kidney function during follow-up.

## Methods

### Design, setting, and study population

A longitudinal study was carried out in Catalonia (Spain), a Mediterranean region of 7,515,398 inhabitants (2012) [21]. The Spanish National Health Service provides universal coverage, financed mainly by tax revenue. The Catalan Health Institute (CHI) manages 285 primary health care centres (PHCCs) in Catalonia that serve 5,501,784 people, corresponding to the 77.2% of the population [22].

**Fig. 1:**
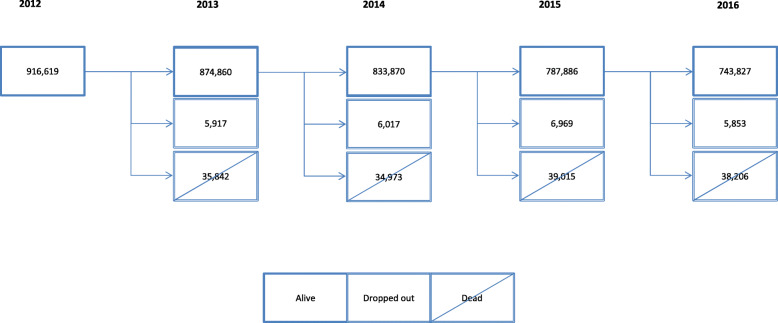
Longitudinal Flow Chart of study period (Years 2012-2016; N=916,619 people)

Inclusion criteria were individuals aged 65–99 years on 31 December 2011 that survived until 31st of December 2012 (index date), with at least one PHCC visit during the 5-year study period (2012–2016). No new entries were allowed in the cohort. Attrition was caused by mortality or transfer to another healthcare provider. We included 916,619 people at baseline and 743,827 completed all follow-up (Fig. 1).

### Data sources

The Information System for Research in Primary Care (SIDIAP) contains the clinical information from the electronic health records (EHR) of the CHI PHCCs since 2006 [23]. The SIDIAP database includes anonymized longitudinal EHR from primary and secondary care that collect information on demographics, symptoms, diagnoses, prescriptions, socio-economic status and the Pharmacy Invoice Registry .

### Variables

All variables were obtained directly from the SIDIAP database [23].

#### Chronic diseases and multimorbidity

In the SIDIAP, diseases are coded in accordance with the International Classification of Diseases, version 10 (ICD-10). An operational definition of multimorbidity was used, i.e., the presence of two or more chronic diseases, based on the selected 60 groups of chronic diseases determined by the Swedish National study of Aging and Care in Kungsholmen [24], with additional clinical, laboratory and medication related parameters for the assessment of certain conditions.

#### Death and drop out

Death was measured as the occurrence of this event, regardless of cause. If a person transferred out of the CHI healthcare provider during the study period, they were considered lost to follow-up (drop-outs).

#### Drugs and polypharmacy

Information on drug exposure was obtained from the Pharmacy Invoice Registry, which includes drugs prescribed by primary care and hospital physicians and subsidized by the national health system. The study included only systemic drugs, and excluded hospital medication, drugs dispensed by hospital pharmacies, drugs not subsidized by public health services and topical medication (e.g. ointments and lotions). Drugs were classified according to the Anatomical Therapeutic Chemical Classification System (ATC) [25]. We used the 4th level (Chemical, Pharmacological or Therapeutic subgroup) to facilitate analysis and interpretation. Chronic use was considered when the person used three packages of the drug during each year of the study period. Each drug was coded into a dichotomous variable. Polypharmacy was defined as the concurrent use of 5 or more drugs in the same individual for each year of the study [7].

#### Kidney function

Kidney function was calculated each follow up year using the four-variable Modification of Diet in Renal Disease (MDRD-4) creatinine calibrated to isotope dilution mass spectrometry (IDMS10) equation to estimate glomerular filtration rate (eGFR) [26]. When one or more eGFR was < 60 ml/min/1.73 m^2^ after the baseline year, we classified it as abnormal kidney function.

#### Liver function

Liver function was determined each follow up year by analysing serum levels of alkaline phosphatase (ALP), alanine transaminase (ALT) and gamma-glutamyl transpeptidase (GGT). Liver function was considered abnormal when after baseline, at least one of the values was as follows: ALP > 2 × 129 IU/L; ALT> 5 × 41 IU/L (men) or ALT> 5 × 33 IU/L (women); GGT > 61 IU/L [27].

#### Other variables

Demographic variables analysed at baseline and at the end of study were: age at baseline (years), sex (men, women), socio-economic status (Mortality in Spanish small areas and Socioeconomic and Environmental Inequalities [MEDEA] index); quintiles from least to most deprived) [28] and number of total visits to PHCCs.

### Statistical analysis

Descriptive statistics were used to summarize overall information. Participants’ characteristics and prevalence of chronic diseases and medication use were measured at baseline and final year, and during the follow-up period.

MP were identified using two steps: 1) the dataset was pre-processed by applying a mixture of Principal Component Analysis and Multiple Correspondence Analysis (PCAmix). For the grouping of participants, it was assumed that the patient population was initially distributed into a fuzzy set of clusters, corresponding to the different MP [29]; 2) In order to model the temporal evolution of individuals and clusters, the sequential individual observations were assumed to follow a dynamic random process represented by a Hidden Markov Model (HMM). The set of HMM parameters was fitted to the observation data by applying the Baum-Welch algorithm. Afterwards, a validation process was applied in order to obtain the optimal parameters and longitudinal clusters. The best cluster trajectory is computed by maximizing the probability of the observed sequence, conditioned to the best computed model parameters (Viterbi Algorithm). Next, we associated each cluster with a hidden state or MP to characterize it.

The final clusters were defined as MP by the research team based on the clinical significance. We eventually obtained ten MP, and also drop out and mortality clusters. To describe the MP, prevalences of diseases in each cluster were calculated. Observed/expected ratios (O/E-ratios) were obtained by dividing disease prevalence in the cluster by disease prevalence in the overall population. Exclusivity, defined as the proportion of patients with the disease included in the cluster over the total number of patients with the disease, was also calculated. A disease was considered to be part of a MP when the O/E-ratio was ≥2. A more detailed description of the applied machine learning technique can be found elsewhere [30].

#### Medication use in multimorbidity patterns

To describe chronic medication use in each multimorbidity pattern (MP), we calculated annual mean packages for each drug in each cluster. Next, we defined O/E-ratios, obtained by dividing mean packages in the cluster by mean packages in the overall population. We also defined exclusivity as the number of medication packages included in the cluster over the total number of medication packages. A medication was considered part of a MP when the O/E-ratio was ≥2. As a complementary approach, we also investigated medicines that presented an exclusivity value higher than the percentage frequency of each cluster.

Logistic regression models were fitted to estimate the association between MP at baseline and abnormal kidney and liver function values during follow up. Odds ratio (OR) and 95% confidence interval (CI) were adjusted for age, sex, socioeconomic status (MEDEA variable) and total number of packages in 2012. We used multiple imputation to minimize the selection bias resulting from the presence of missing values for MEDEA (7%). We used multiple imputation by chained equations to obtain seven imputed datasets [31]. The final models were fitted with multiple imputed datasets using Rubin’s rules to combine effect estimates and standard errors to allow for the uncertainty related to missing data.

The analyses were carried out using R version 3.6.1 (R Foundation for Statistical Computing, Vienna, Austria). The significance level was set at α = 0.05.

## Results

A total of 916,619 people over 65 years of age living in Catalonia were included at baseline (women: 57.8%; mean age: 75.4; standard deviation [SD]: 7.4), 853,085 (93.1%) met multimorbidity criteria, and 487,502 (53.2%) criteria for polypharmacy. At the end of the study period, 743,827 participants had completed follow-up (Fig. 1). The mean number of chronic diseases was 6 (interquartile range [IQR] 4.0–8.0) at baseline and 7 (IQR 5.0–10.0) in the final year, whereas mean number of medicines during follow-up was 5 (IQR baseline 2.0–8.0 and IQR at final year 3.0–8.0). The proportion of patients with multimorbidity and polypharmacy was higher at the end of the study period than at baseline (93.1 and 53.3% vs 97.2 and 56.8%, respectively) (Table 1).

**Table 1:** Variables characterizing each cluster in the study at baseline and at the end of the study (*N* = 916,619)

Variables	Cluster 1 - Non-Specific	Cluster 2 - Eye Impairment and Mental	Cluster 3 - Minority Metabolic Autoimmune-Inflammatory	Cluster 4 - Cardio-Circulatory and Renal	Cluster 5 - Cardio-Circulatory, Mental, Respiratory and Genitourinary	Cluster 6 - Neurological, Digestive and Circulatory	Cluster 7 - Respiratory and Ear	Cluster 8 - Digestive	Cluster 9 - Neurological, Musculoskeletal and minor	Cluster 10 - Multisystem Pattern	Overall (patients alive)
	N_2012_=384787 (42.0%) - N_2016_=258700 (34.8%)	N_2012_=177087 (19.3%) - N_2016_=155008 (20.8%)	N_2012_=72190 (7.9%) - N_2016_=73237 (9.8%)	N_2012_=60192 (6.6%) - N_2016_=46244 (6.2%)	N_2012_=54324 (5.9%) - N_2016_=45463 (6.1%)	N_2012_=42422 (4.6%) - N_2016_=41120 (5.5%)	N_2012_=41644 (4.5%) - N_2016_=38961 (5.2%)	N_2012_=35692 (3.9%) - N_2016_=31744 (4.3%)	N_2012_=33283 (3.6%) - N_2016_=36522 (4.9%)	N_2012_=14998 (1.6%) - N_2016_=16828 (2.3%)	N_2012_=916619 - N_2016_= 743827
**Sex:**											
Female	201958 (52.5%) - 137931 (53.3%)	130572 (73.7%) - 113574 (73.3%)	31395 (43.5%) - 31490 (43.0%)	42638 (70.8%) - 32786 (70.9%)	3618 (6.7%) - 2902 (6.4%)	23577 (55.6%) - 22601 (55.0%)	31143 (74.8%) - 28838 (74.0%)	19915 (55.8%) - 17846 (56.2%)	30830 (92.6%) - 33718 (92.3%)	13485 (89.9%) - 15005 (89.2%)	529131 (57.8%) - 436691 (58.7%)
Male	182829 (47.5%) - 120769 (46.7%)	46515 (26.3%) - 41434 (26.7%)	40795 (56.5%) - 41747 (57.0%)	17554 (29.2%) - 13458 (29.1%)	50706 (93.3%) - 42561 (93.6%)	18845 (44.4%) - 18519 (45.0%)	10501 (25.2%) - 10123 (26.0%)	15777 (44.2%) - 13898 (43.8%)	2453 (7.4%) - 2804 (7.7%)	1513 (10.1%) - 1823 (10.8%)	387488 (42.2%) - 307136 (41.3%)
**Patients with Multimorbidity:**	323153 (84.0%) - 238466 (92.2%)	176734 (99.8%) - 154922 (99.9%)	71562 (99.1%) - 73039 (99.7%)	60192 (100.0%) - 46243 (100.0%)	54318 (100.0%) - 45463 (100.0%)	42126 (99.3%) - 41020 (99.8%)	41413 (99.4%) - 38885 (99.8%)	35306 (98.9%) - 31628 (99.6%)	33283 (100.0%) - 36522 (100.0%)	14998 (100.0%) - 16828 (100.0%)	853085 (93.1%) - 723016 (97.2%)
**Patients with Polypharmacy:**	122956 (32.0%) - 84206 (32.5%)	110504 (62.4%) - 99185 (64.0%)	43115 (59.7%) - 44500 (60.8%)	51759 (86.0%) - 40528 (87.6%)	43829 (80.7%) - 38159 (83.9%)	30644 (72.2%) - 29788 (72.4%)	29416 (70.6%) - 28254 (72.5%)	20336 (57.0%) - 18717 (59.0%)	22739 (68.3%) - 25338 (69.4%)	12204 (81.4%) - 13710 (81.5%)	487502 (53.2%) - 422385 (56.8%)
**Age (Mean):**	74.8 - 73.3	74.2 - 73.5	75.9 - 74.5	80.6 - 78.3	76.7 - 75.0	78.7 - 76.3	75.3 - 74.0	74.8 - 73.6	72.8 - 72.5	76.9 - 75.8	75.5 - 74.1
**Age (n,%):**											
[65,70)	121226 (31.5%) - 96106 (37.1%)	55687 (31.4%) - 52749 (34.0%)	17825 (24.7%) - 21292 (29.1%)	4711 (7.8%) - 5461 (11.8%)	10906 (20.1%) - 11395 (25.1%)	5578 (13.1%) - 7904 (19.2%)	10981 (26.4%) - 12061 (31.0%)	10135 (28.4%) - 10472 (33.0%)	12210 (36.7%) - 13773 (37.7%)	2919 (19.5%) - 3788 (22.5%)	252178 (27.5%) - 235001 (31.6%)
[70,80)	162009 (42.1%) - 113532 (43.9%)	81567 (46.1%) - 72827 (47.0%)	31466 (43.6%) - 34163 (46.6%)	20490 (34.0%) - 20320 (43.9%)	24074 (44.3%) - 22198 (48.8%)	16968 (40.0%) - 19730 (48.0%)	19002 (45.6%) - 18753 (48.1%)	16359 (45.8%) - 15101 (47.6%)	16152 (48.5%) - 17933 (49.1%)	6499 (43.3%) - 7863 (46.7%)	394586 (43.1%) - 342420 (46%)
[80,90)	86495 (22.5%) - 44749 (17.3%)	36744 (20.7%) - 27946 (18.0%)	20059 (27.8%) - 16495 (22.5%)	29143 (48.4%) - 18693 (40.4%)	17052 (31.4%) - 11113 (24.4%)	16937 (39.9%) - 12481 (30.4%)	10335 (24.8%) - 7616 (19.5%)	8316 (23.3%) - 5851 (18.4%)	4747 (14.3%) - 4682 (12.8%)	4916 (32.8%) - 4798 (28.5%)	234744 (25.6%) - 154424 (20.8%)
[90,99]	15057 (3.9%) - 4313 (1.7%)	3089 (1.7%) - 1486 (1.0%)	2840 (3.9%) - 1287 (1.8%)	5848 (9.7%) - 1770 (3.8%)	2292 (4.2%) - 757 (1.7%)	2939 (6.9%) - 1005 (2.4%)	1326 (3.2%) - 531 (1.4%)	882 (2.5%) - 320 (1.0%)	174 (0.5%) - 134 (0.4%)	664 (4.4%) - 379 (2.3%)	35111 (3.8%) - 11982 (1.6%)
**MEDEA:**											
R	82025 (22.7%) - 54244 (21.6%)	33553 (20.0%) - 29758 (19.8%)	15314 (22.7%) - 15667 (22.0%)	12276 (23.5%) - 9279 (20.7%)	9282 (19.1%) - 7490 (17.1%)	8888 (23.7%) - 8089 (20.4%)	7512 (19.4%) - 7006 (18.6%)	6396 (19.8%) - 5650 (18.4%)	4888 (15.4%) - 5456 (15.4%)	2115 (15.3%) - 2350 (14.5%)	182249 (21.4%) - 144989 (20.1%)
U1	67060 (18.6%) - 47089 (18.8%)	26625 (15.9%) - 23782 (15.8%)	10987 (16.3%) - 11839 (16.6%)	8247 (15.8%) - 7255 (16.2%)	7144 (14.7%) - 6673 (15.2%)	6691 (17.8%) - 7247 (18.3%)	5657 (14.6%) - 5535 (14.7%)	5534 (17.1%) - 5400 (17.6%)	4306 (13.5%) - 4983 (14.1%)	2540 (18.4%) - 2975 (18.3%)	144791 (17.0%) - 122778 (17.0%)
U2	58542 (16.2%) - 41383 (16.5%)	27027 (16.1%) - 24586 (16.3%)	10976 (16.3%) - 11809 (16.6%)	8048 (15.4%) - 7160 (16.0%)	7442 (15.3%) - 7043 (16.0%)	5862 (15.6%) - 6555 (16.6%)	6062 (15.6%) - 5999 (15.9%)	5202 (16.1%) - 5056 (16.4%)	4964 (15.6%) - 5641 (15.9%)	2306 (16.7%) - 2806 (17.3%)	136431 (16.0%) - 118038 (16.4%)
U3	57967 (16.0%) - 40622 (16.2%)	27628 (16.5%) - 25111 (16.7%)	10665 (15.8%) - 11607 (16.3%)	8076 (15.5%) - 7179 (16.0%)	8077 (16.6%) - 7486 (17.1%)	6116 (16.3%) - 6568 (16.6%)	6409 (16.5%) - 6301 (16.7%)	5192 (16.1%) - 5090 (16.6%)	5745 (18.1%) - 6362 (18.0%)	2347 (17.0%) - 2796 (17.2%)	138222 (16.2%) - 119122 (16.5%)
U4	52828 (14.6%) - 37452 (14.9%)	27931 (16.6%) - 25006 (16.6%)	10526 (15.6%) - 10980 (15.4%)	7832 (15.0%) - 6990 (15.6%)	8361 (17.2%) - 7777 (17.7%)	5329 (14.2%) - 5952 (15.0%)	6730 (17.4%) - 6644 (17.6%)	5194 (16.1%) - 4974 (16.2%)	6003 (18.9%) - 6580 (18.6%)	2254 (16.3%) - 2724 (16.8%)	132988 (15.6%) - 115079 (16.0%)
U5	42956 (11.9%) - 30178 (12.0%)	25050 (14.9%) - 22140 (14.7%)	8855 (13.2%) - 9253 (13.0%)	7775 (14.9%) - 6880 (15.4%)	8236 (17.0%) - 7432 (16.9%)	4686 (12.5%) - 5164 (13.0%)	6378 (16.5%) - 6282 (16.6%)	4805 (14.9%) - 4572 (14.9%)	5894 (18.5%) - 6379 (18.0%)	2248 (16.3%) - 2593 (16.0%)	116883 (13.7%) - 100873 (14.0%)
**N. Chronic diseases, median [IQR]:**	4.0 [2.0;5.0] - 5.0 [3.0;6.0]	6.0 [5.0;8.0] - 8.0 [6.0;9.0]	7.0 [5.0;8.0] - 8.0 [6.0;10.0]	9.0 [7.0;11.0] - 10.0 [8.0;12.0]	8.0 [7.0;10.0] - 10.0 [8.0;12.0]	7.0 [5.0;9.0] - 9.0 [6.0;11.0]	7.0 [5.0;9.0] - 9.0 [7.0;11.0]	7.0 [5.0;9.0] - 8.0 [6.0;11.0]	9.0 [7.0;10.0] - 10.0 [9.0;12.0]	11.0 [10.0;13.0] - 13.0 [11.0;15.0]	6.0 [4.0;8.0] - 7.0 [5.0;10.0]
**N. Chronic diseases (n,%):**											
0	25380 (6.6%) - 6584 (2.5%)	0 (0.0%) - 0 (0.0%)	0 (0.0%) - 0 (0.0%)	0 (0.0%) - 0 (0.0%)	0 (0.0%) - 0 (0.0%)	0 (0.0%) - 0 (0.0%)	0 (0.0%) - 0 (0.0%)	0 (0.0%) - 0 (0.0%)	0 (0.0%) - 0 (0.0%)	0 (0.0%) - 0 (0.0%)	25380 (2.8%) - 6584 (0.9%)
1	36254 (9.4%) - 13650 (5.3%)	353 (0.2%) - 86 (0.1%)	628 (0.9%) - 198 (0.3%)	0 (0.0%) - 1 (0.0%)	6 (0.0%) - 0 (0.0%)	296 (0.7%) - 100 (0.2%)	231 (0.6%) - 76 (0.2%)	386 (1.1%) - 116 (0.4%)	0 (0.0%) - 0 (0.0%)	0 (0.0%) - 0 (0.0%)	38154 (4.2%) - 14227 (1.9%)
[ 2, 5)	201282 (52.3%) - 108605 (42.0%)	30742 (17.4%) - 11516 (7.4%)	13785 (19.1%) - 7640 (10.4%)	1809 (3.0%) - 549 (1.2%)	1974 (3.6%) - 506 (1.1%)	6032 (14.2%) - 3276 (8.0%)	5751 (13.8%) - 2428 (6.2%)	6751 (18.9%) - 3105 (9.8%)	698 (2.1%) - 173 (0.5%)	12 (0.1%) - 1 (0.0%)	268836 (29.3%) - 137799 (18.5%)
[ 5,10)	120673 (31.4%) - 126309 (48.8%)	132238 (74.7%) - 111530 (72.0%)	47338 (65.6%) - 44773 (61.1%)	32584 (54.1%) - 17095 (37.0%)	34571 (63.6%) - 19151 (42.1%)	25615 (60.4%) - 21911 (53.3%)	25450 (61.1%) - 20204 (51.9%)	21261 (59.6%) - 17305 (54.5%)	20365 (61.2%) - 13811 (37.8%)	3614 (24.1%) - 1583 (9.4%)	463709 (50.6%) - 393672 (52.9%)
≥10	1198 (0.3%) - 3552 (1.4%)	13754 (7.8%) - 31876 (20.6%)	10439 (14.5%) - 20626 (28.2%)	25799 (42.9%) - 28599 (61.8%)	17773 (32.7%) - 25806 (56.8%)	10479 (24.7%) - 15833 (38.5%)	10212 (24.5%) - 16253 (41.7%)	7294 (20.4%) - 11218 (35.3%)	12220 (36.7%) - 22538 (61.7%)	11372 (75.8%) - 15244 (90.6%)	120540 (13.2%) - 191545 (25.8%)
**N. of drugs, median [IQR]:**	3.0 [1.0;5.0] - 3.0 [1.0;5.0]	6.0 [3.0;8.0] - 6.0 [4.0;8.0]	5.0 [3.0;8.0] - 5.0 [3.0;8.0]	8.0 [6.0;11.0] - 8.0 [6.0;11.0]	8.0 [5.0;10.0] - 6.0 [8.0;10.0]	7.0 [4.0;10.0] - 7.0 [4.0;9.0]	7.0 [4.0;10.0] - 7.0 [4.0;10.0]	5.0 [3.0;8.0] - 5.0 [3.0;8.0]	6.0 [4.0;9.0] - 6.0 [4.0;9.0]	8.0 [5.0;11.0] - 8.0 [5.0;10.0]	5.0 [2.0;8.0] - 5.0 [3.0;8.0]
**N. of drugs (n,%):**											
0	82698 (21.5%) - 41989 (16.2%)	9688 (5.5%) - 5355 (3.5%)	5408 (7.5%) - 3759 (5.1%)	2540 (4.2%) - 761 (1.6%)	2186 (4.0%) - 800 (1.8%)	2815 (6.6%) - 1463 (3.6%)	2366 (5.7%) - 1293 (3.3%)	3597 (10.1%) - 2199 (6.9%)	1473 (4.4%) - 1068 (2.9%)	597 (4.0%) - 265 (1.6%)	113368 (12.4%) - 58952 (7.9%)
1	39683 (10.3%) - 28971 (11.2%)	6639 (3.7%) - 5728 (3.7%)	3467 (4.8%) - 3626 (5.0%)	505 (0.8%) - 353 (0.8%)	929 (1.7%) - 624 (1.4%)	1161 (2.7%) - 1257 (3.1%)	1246 (3.0%) - 1222 (3.1%)	2083 (5.8%) - 1822 (5.7%)	1159 (3.5%) - 1305 (3.6%)	210 (1.4%) - 283 (1.7%)	57082 (6.2%) - 45191 (6.1%)
[ 2, 5)	139450 (36.2%) - 103534 (40.0%)	50256 (28.4%) - 44740 (28.9%)	20200 (28.0%) - 21352 (29.2%)	5388 (9.0%) - 4602 (10.0%)	7380 (13.6%) - 5880 (12.9%)	7802 (18.4%) - 8612 (20.9%)	8616 (20.7%) - 8192 (21.0%)	9676 (27.1%) - 9006 (28.4%)	7912 (23.8%) - 8811 (24.1%)	1987 (13.2%) - 2570 (15.3%)	258667 (28.2%) - 217299 (29.2%)
[ 5,10)	108886 (28.3%) - 76263 (29.5%)	88542 (50.0%) - 79758 (51.5%)	32682 (45.3%) - 34143 (46.6%)	28606 (47.5%) - 23462 (50.7%)	26833 (49.4%) - 23058 (50.7%)	19941 (47.0%) - 20083 (48.8%)	18623 (44.7%) - 18442 (47.3%)	14879 (41.7%) - 13799 (43.5%)	16599 (49.9%) - 18510 (50.7%)	7090 (47.3%) - 8278 (49.2%)	362681 (39.6%) - 315796 (42.5%)
≥10	14070 (3.7%) - 7943 (3.1%)	21962 (12.4%) - 19427 (12.5%)	10433 (14.5%) - 10357 (14.1%)	23153 (38.5%) - 17066 (36.9%)	16996 (31.3%) - 15101 (33.2%)	10703 (25.2%) - 9705 (23.6%)	10793 (25.9%) - 9812 (25.2%)	5457 (15.3%) - 4918 (15.5%)	6140 (18.4%) - 6828 (18.7%)	5114 (34.1%) - 5432 (32.3%)	124821 (13.6%) - 106589 (14.3%)
**N. of visits, median [IQR]:**	7.0 [3.0;12.0] - 7.0 [3.0;12.0]	10.0 [6.0;16.0] - 10.0 [6.0;16.0]	10.0 [6.0;17.0] - 10.0 [6.0;18.0]	20.0 [11.0;32.0] - 22.0 [11.0;34.0]	14.0 [9.0;23.0] - 15.0 [9.0;25.0]	12.0 [7.0;20.0] - 12.0 [7.0;21.0]	12.0 [7.0;20.0] - 12.0 [7.0;20.0]	11.0 [6.0;19.0] - 11.0 [6.0;19.0]	12.0 [8.0;19.0] - 12.0 [8.0;19.0]	15.0 [9.0;23.0] - 15.0 [9.0;24.0]	9.0 [5.0;16.0] - 10.0 [5.0;17.0]
**N. of visits (n,%):**											
0	36975 (9.6%) - 17397 (6.7%)	3722 (2.1%) - 2380 (1.5%)	1839 (2.5%) - 1335 (1.8%)	863 (1.4%) - 390 (0.8%)	763 (1.4%) - 395 (0.9%)	1147 (2.7%) - 780 (1.9%)	779 (1.9%) - 544 (1.4%)	1205 (3.4%) - 749 (2.4%)	390 (1.2%) - 311 (0.9%)	262 (1.7%) - 131 (0.8%)	47945 (5.2%) - 24412 (3.3%)
1	23812 (6.2%) - 14629 (5.7%)	3649 (2.1%) - 3624 (2.3%)	1830 (2.5%) - 1922 (2.6%)	649 (1.1%) - 498 (1.1%)	629 (1.2%) - 568 (1.2%)	1051 (2.5%) - 1013 (2.5%)	749 (1.8%) - 672 (1.7%)	1043 (2.9%) - 866 (2.7%)	315 (0.9%) - 417 (1.1%)	157 (1.0%) - 187 (1.1%)	33884 (3.7%) - 24396 (3.3%)
[ 2, 5)	77936 (20.3%) - 54464 (21.1%)	20693 (11.7%) - 18928 (12.2%)	9076 (12.6%) - 9669 (13.2%)	3064 (5.1%) - 2534 (5.5%)	3739 (6.9%) - 3050 (6.7%)	4606 (10.9%) - 4510 (11.0%)	3793 (9.1%) - 3558 (9.1%)	4310 (12.1%) - 3938 (12.4%)	2386 (7.2%) - 2866 (7.8%)	836 (5.6%) - 1104 (6.6%)	130439 (14.2%) - 104621 (14.1%)
[ 5, 10)	117379 (30.5%) - 82678 (32.0%)	52731 (29.8%) - 46906 (30.3%)	20119 (27.9%) - 20721 (28.3%)	8287 (13.8%) - 5948 (12.9%)	10988 (20.2%) - 9136 (20.1%)	9698 (22.9%) - 9891 (24.1%)	10028 (24.1%) - 9391 (24.1%)	9029 (25.3%) - 8329 (26.2%)	8278 (24.9%) - 8991 (24.6%)	2812 (18.7%) - 3234 (19.2%)	249349 (27.2%) - 205225 (27.6%)
≥10	128685 (33.4%) - 89532 (34.6%)	96292 (54.4%) - 83170 (53.7%)	39326 (54.5%) - 39590 (54.1%)	47329 (78.6%) - 36874 (79.7%)	38205 (70.3%) - 32314 (71.1%)	25920 (61.1%) - 24926 (60.6%)	26295 (63.1%) - 24796 (63.6%)	20105 (56.3%) - 17862 (56.3%)	21914 (65.8%) - 23937 (65.5%)	10931 (72.9%) - 12172 (72.3%)	455002 (49.7%) - 385173 (51.8%)

The most prevalent invoiced drugs, from highest to lowest, were proton pump inhibitors (PPI), 3-hidroxi-3-metil-glutaril-CoA reductase (HMGCoA-reductase) inhibitors and anilides. During follow-up, the most common drugs remained unchanged, but the prescription had expanded to include more patients (Supplementary File 1).

Ten MP were identified at baseline (see characteristics of each MP in Table 1). Except for Cluster 1 (C1) and Cluster 4 (C4), the number of people included in all clusters increased during the study period. Most people remain in the same cluster during follow up. For all MP, the most common shift is dying. The probability of dying varies significantly depending on which MP the patient was classified.

### Characteristics of medication use in relation to multimorbidity patterns

**Fig. 2:**
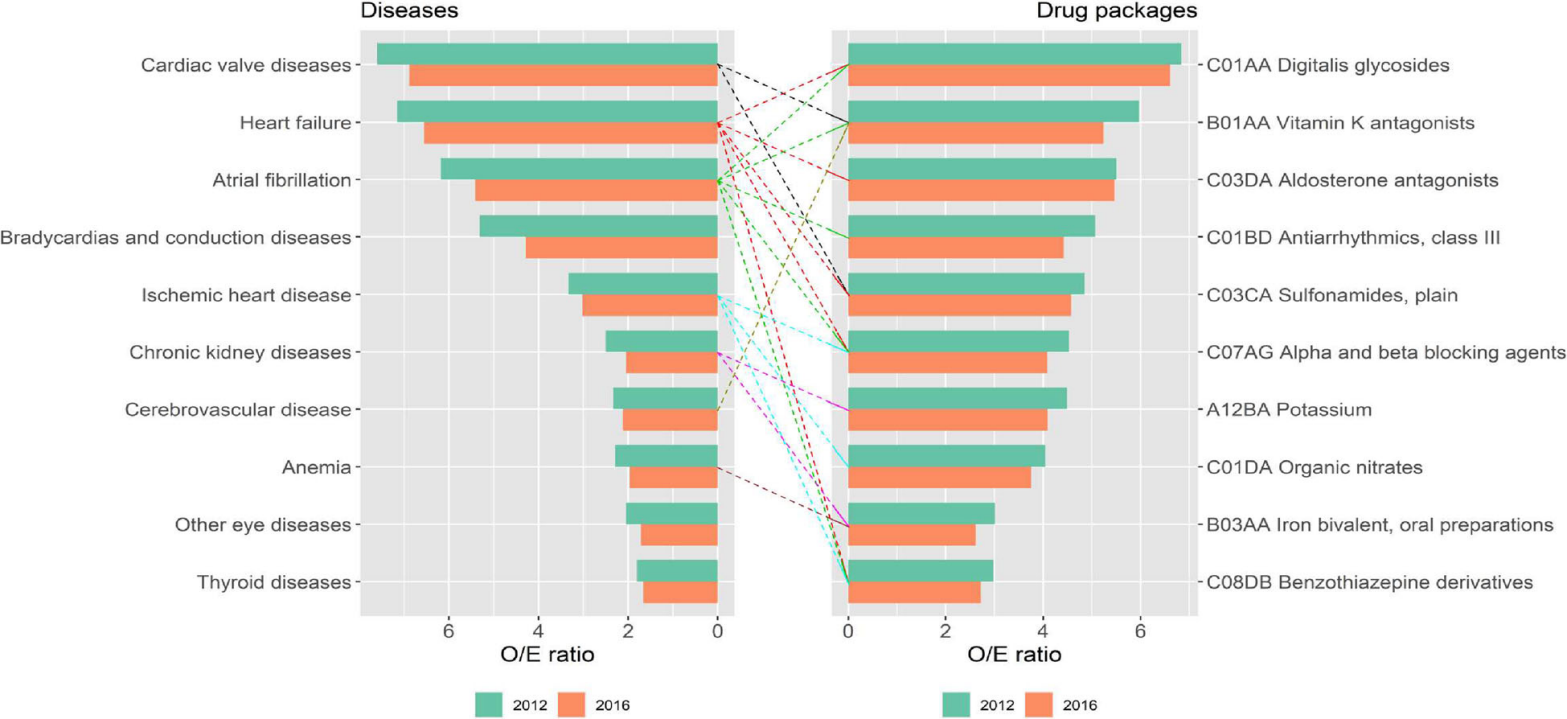
Cardio-Circulatory and Renal patterns in relation with drug prescription

Overall, we observed that overrepresented (O/E-ratio > 2) drugs (measured as mean packages) in each MP were associated with the overrepresented diseases in that same MP. Naturally, these drugs are intended to treat specific chronic diseases overrepresented in their corresponding MP (Supplementary File 2). For example, in:

- *Cluster2 (C2)-Eye Impairment and Mental*: The most used drugs at baseline were ophthalmologic beta blockers. It should be noted that more than half of the general population with glaucoma and under treatment with ophthalmic beta blockers are included in this cluster (Exclusivity 50.59 and 48.75% at baseline, respectively).
- *Cluster3 (C3)-Minority Metabolic Autoimmune-Inflammatory*: At baseline, the most overrepresented drugs were glucocorticoids, uric acid lowering agents, and Vitamin D and analogues.
- *Cluster4 (C4)-Cardio-Circulatory and Renal*: At baseline, people included in this pattern had 17 different drug groups overrepresented, mainly to treat ischemic cardiovascular disease, heart failure, valvular heart disease, cardiac arrhythmias, diabetes, kidney failure and anaemia (Fig. 2).
- *Cluster5 (C5)-Cardio-Circulatory, Mental, Respiratory and Genitourinary*: At baseline, people included in this pattern had 20 different drug groups overrepresented: peripheral vasodilators, bronchodilators, drugs for ischemic cardiomyopathy, heart failure, prostate diseases and diabetes.
- *Cluster6 (C6)-Neurological, Digestive and Circulatory*: Overrepresentation in this cluster is mostly constituted by patients with Parkinson’s disease and corresponding treatment, mainly dopamine, dopamine derivatives and other medicines used in neurological disorders. These diseases and related medications are practically exclusive to this cluster (more than 90.0% at the beginning of the study).
- *Cluster7 (C7)-Respiratory and Ear*: Drugs included in this cluster were glucocorticoids, adrenergics in combination with corticosteroids or other drugs excluding anticholinergics, selective beta-2-adrenoreceptor agonists, anticholinergics, corticosteroids, and antihistamines for systemic use.
- *Cluster8 (*C8)-Digestive: The most prescribed medication corresponds to drugs used for the treatment of chronic liver diseases.
- *Cluster9 (*C9)-Neurological, Musculoskeletal and Minor: In this group the most overrepresented drugs are anti-inflammatory and analgesics, in agreement with the MP.
- *Cluster10 (*C10)-Multisystem: The least common pattern, it has multiple overrepresented disorders from different systems, and the corresponding medications used to treat these disorders.

**Fig. 3:**
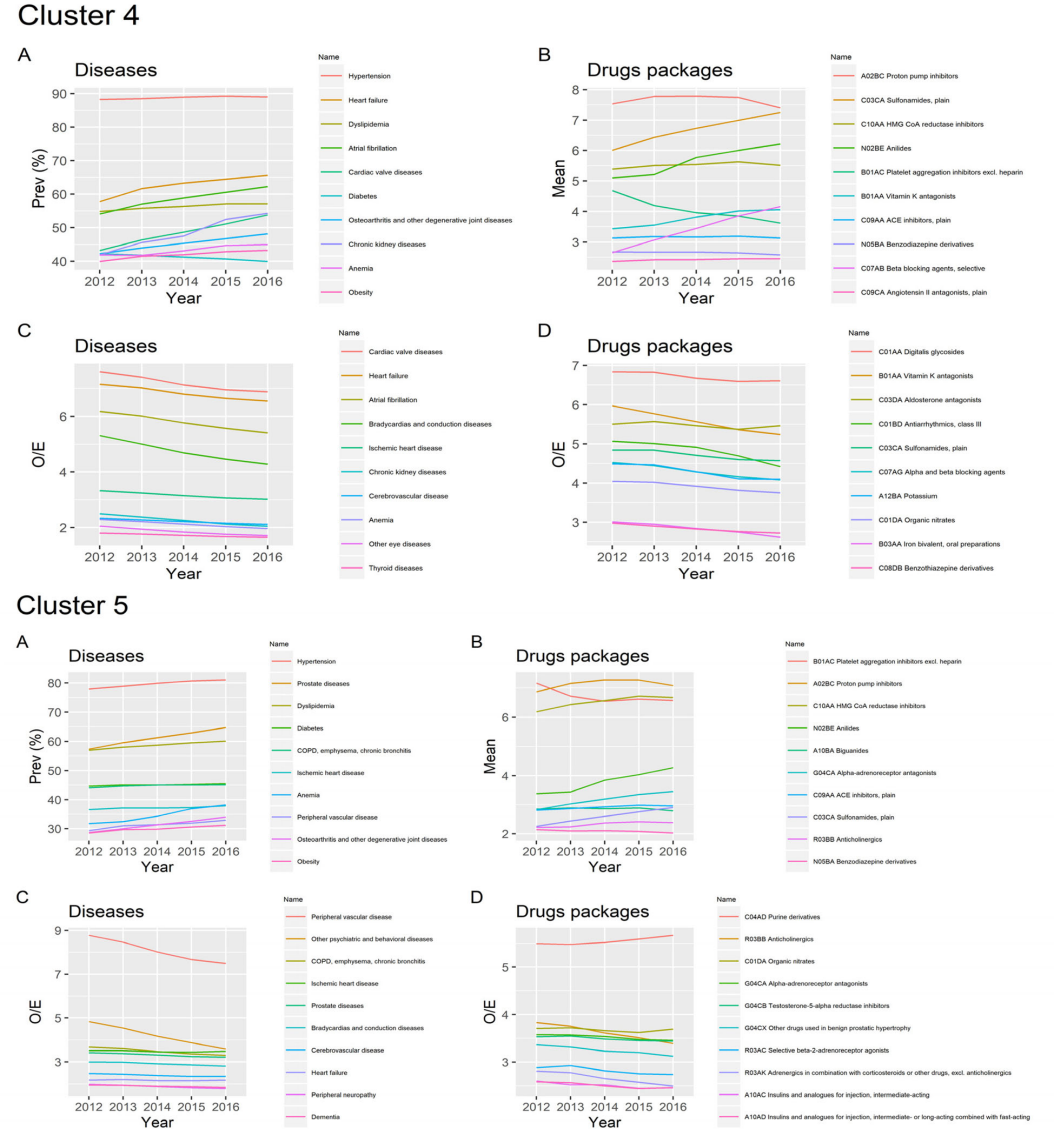
Prevalence and O/E ratio evolution over time for diseases and drugs in Cluster 4 - Cardio Circulatory and Renal and Cluster 5 - Cardio Circulatory, Mental, Respiratory and Genitourinary

As an example, Fig. 3 represents the evolution during follow up of the O/E-ratio and mean number of medication packages for the ten most used drugs in *C4-Cardio-Circulatory and Renal* and *C5-Cardio-Circulatory, Mental, Respiratory and Genitourinary*. In these two MP, both the O/E-ratio and mean number of medication packages increase or remain stable over time.

Considering *C1-Non-Specific* as a reference*,* an increase in abnormal kidney function was observed in the different MP over the study period. The highest proportion of abnormal kidney function was observed in the C4-Cardio-Circulatory and Renal (OR 2.19; CI 95% 2.15–2.23) and C3-Minority Metabolic Autoimmune-Inflammatory MP (OR 2.16; CI 95% 2.12–2.20) (Table 2 and Supplementary file 3). The MP with a higher risk of abnormal liver function were C8-Digestive (OR 3.39; CI 95% 3.30–3.49), followed by C4-Cardio-Circulatory and Renal (OR 1.96; CI 95% 1.91–2.02) (Table 2 and Supplementary File 4).

**Table 2:**
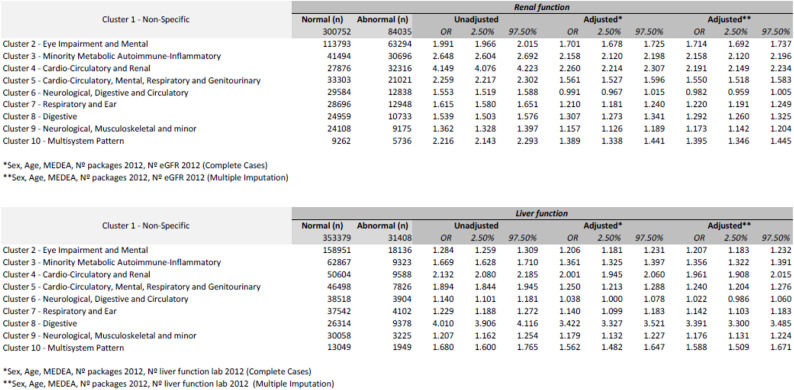
Logistic regression models for Kidney and Liver function by cluster.

## Discussion

### Key results

This study informs on the use of medication by the elderly population during 5 years of follow up, according to the ten most common MP. Predictably, the most overrepresented drugs in each MP coincide with the most overrepresented disorders in that same MP. Also, the medicines most prescribed in the study population remain unchanged throughout the follow up period. The analysis of polypharmacy based on specific MP and their association with abnormal liver and kidney function has revealed that the patients included in the various MP present high rates of abnormal liver and kidney function when compared to the MP *C1-Non-Specific*, underlining the need for new safety criteria in these patients [32, 33].

### Polypharmacy and their evolution

In *C2-Eye Impairment and Mental*, the medicines most frequently prescribed correspond to the ocular disorders diagnosed. However, in other groups of diseases of this MP, for instance the *Neurotic, stress-related and somatoform diseases*, which correspond mainly to cigarette consumption, the pharmacological treatment is not subsidised by the public health system and consequently does not appear in the Pharmacy Invoice Registry.

In *C3-Minority Metabolic Autoimmune-Inflammatory*, over 50% of diseases are exclusive of this cluster. However, even if the medication is associated with overrepresented conditions, it does not have high exclusivity since these medicines have different authorised indications, for instance glucocorticoids and Vitamin D and analogues, which are even indicated off-label [34, 35].

The MP *C4-Cardio-Circulatory and Renal* and *C5-Cardio-Circulatory, Mental, Respiratory and Genitourinary*, illustrate the patients with multimorbidity who are prescribed polypharmacy to treat overrepresented diseases. In addition, some diseases as diabetes that are not overrepresented might need various medicines to control the condition. Moreover, recent intensification of treatments has translated into an increase in drug prescription [36, 37].

In *C9-Neurological, Musculoskeletal and Minor*, metamizole has replaced traditional nonsteroidal anti-inflammatory drugs (NSAIDs), probably due to emerging safety concerns of NSAIDs regarding the kidney [38], cardiovascular [39, 40], and digestive systems [39].

### Abnormal kidney and liver failure function

This study shows that most patients with multimorbidity receiving polypharmacy present abnormal kidney and liver function. While the design of this study cannot explain the causes of the abnormal kidney and liver function observed, we hypothesise that they probably originate from the baseline disorders of the patients, adverse effects from the medication, inappropriate prescribing and lack of adjustment to the kidney and liver function of each patient, which are usually affected by old age [11–13, 41–43]. This study, based on medication packages dispensed in pharmacies, underlines the importance of stringent monitoring of prescriptions, particularly in patients that might have abnormal kidney and liver function. We recommend the use of prompts in the EHR to adjust medication dosage in accordance with liver function and eGFR.

Abnormal kidney function is highest in patients from MP C4 and C3. In MP *C4 -Cardio-Circulatory and Renal*, in addition to the baseline kidney and cardiovascular disorders of these patients, abnormal kidney function has been attributed to the hemodynamic effects of diuretics causing nephrotoxicity [41]. The MP *C3-Minority Metabolic Autoimmune-Inflammatory* includes patients with hypothyroidism [44] and overrepresentation of allopurinol, both causes of abnormal kidney function.

Abnormal liver function is highest in patients from clusters C8 and C4. The risk is highest in patients in *C8 - Digestive*, since this pattern represents patients with liver disease. *C4- Cardio-Circulatory and Renal* has the oldest patients and the highest prescription of vitamin K antagonists, which can cause cholestasis [42].

### Comparison with the literature

Most studies on MP use a cross-sectional design. Some publications include longitudinal data, but to our knowledge no data on the association of MP and abnormal kidney and liver function have been published [45].. In the literature, European articles underscore medication for depression and chronic obstructive pulmonary disease (COPD) in the elderly [18], which we included in clusters C9 and C5, respectively. In contrast, Japanese authors observe the highest risk of polypharmacy in malignant, digestive and urologic patterns [17]. While we excluded drugs for the treatment of malignancies in our study, we did not observe overrepresentation of drugs for the gastrointestinal system, since they are the type of medication most consumed in the general population, nor for urological diseases, which are highly prevalent in the population over 65 years. Interestingly, in the Japanese study only 25% of patients were over 65 years of age. Ultimately, if we analysed the drugs overrepresented in specific patterns such as the cardiovascular (C4 and C5), these medicines would practically replicate polypharmacy patterns described in other publications [18], i.e., drugs for cardiovascular diseases, for diabetes and for gout. However, our medication patterns included also the treatments for other diseases in MP C4 and C5, for instance anaemia, pain, glaucoma, COPD and benign prostatic hyperplasia.

### Strengths and limitations

One of the major strengths of this study is the use of a large, high-quality database that originates from the primary care EHR, which includes a large proportion of the population with multimorbidity and with polypharmacy [23]. Furthermore, we have used a classification for chronic diseases previously validated by a clinically driven methodology, which allows a homogeneous assessment of chronic diseases and polypharmacy in a manageable number of categories, and also the uniform evaluation of chronicity in the European Union [24].

This study also presents some limitations. Firstly, we only considered the medications for which at least three packages during each year of the study period had been dispensed. While this could underestimate some medicines, it is extremely unusual to dispense less than 3 packages per year of drugs treating chronic diseases. Similarly, we excluded the medication for acute conditions, some of which can cause temporary abnormalities in kidney and liver function. Secondly, the SIDIAP only collects information on drugs prescribed by primary care and hospital physicians which are dispensed by community pharmacies. Consequently, our analysis cannot rule out active ingredients included in hospital drug regimes, over-the-counter medicines and para-pharmacy products, which might also impair kidney and liver function. Thirdly and finally, censoring of people who died and people who transferred might have influenced estimations of risk associations in a competing-risk scenario, which was not an objective of this study.

Our research shows that while prescription of polypharmacy might be justified with regard to clinical guidelines, it increases the risk of adverse drug reactions and might negatively affect kidney and liver function. Crucially, the risk of overtreatment, which occurs when the prescribed medications have no clinically significant benefit and when the risk of adverse effects associated with an additional medication outweighs the overall benefit of the treatment, is high in polypharmacy [46, 47].

## Conclusions

Polypharmacy is highly prevalent in older adults. The most frequently prescribed medicines were related to MP and their consumption was maintained throughout the follow-up period.

Finally, this study provides real-world data on the evolution of polypharmacy and multimorbidity among older adults and describes the association between polypharmacy and abnormal liver and kidney function in this population.

## Supplementary information

**Additional file 1.** Prevalence of Top 30 Drugs during the follow up period in people over 65 years in Catalonia.

**Additional file 2.** Multimorbidity patterns with Hidden Markov models at baseline (year 2012) and final year (2016) of the study. Drug use

**Additional file 3.** Evolution of kidney function during follow up by clusters.

**Additional file 4.** Evolution of liver function during follow up by clusters.

## Data Availability

The datasets are not available, as researchers have signed an agreement with the Information System for the Development of Research in Primary Care (SIDIAP) concerning confidentiality and security of the dataset, which forbids providing data to third parties. The SIDIAP is subject to periodic audits.

## Abbreviations

ALP: Alkaline phosphatase
ALT: Alanine transaminase
ATC: Anatomical therapeutic chemical classification system
C1: Cluster 1
C2: Cluster 2
C3: Cluster 3
C4: Cluster 4
C5: Cluster 5
C6: Cluster 6
C7: Cluster7
C8: Cluster 8
C9: Cluster 9
C10: Cluster 10
CHI: Catalan health institute
CI: Confidence interval
COPD: Chronic obstructive pulmonary disease
eGFR: Estimate glomerular filtration rate
EHR: Electronic health records
GGT: Gamma-glutamyl transpeptidase
HMGCoA-reductase: 3-Hidroxi-3-metil-glutaril-CoA reductase
HMM: Hidden markov model
ICD-10: International classification of diseases, version 10
IDMS10: Creatinine calibrated to isotope dilution mass spectrometry
IQR: Interquartile range
MEDEA: Mortality in Spanish small areas and socioeconomic and environmental inequalities
MDRD-4: Four-variable modification of diet in renal disease
MP: Multimorbidity pattern
NSAIDs: Nonsteroidal anti-inflammatory drugs
O/E-ratios: Observed/expected ratios
OR: Odds ratio
PCAmix: Principal component analysis and multiple correspondence analysis
PHCCs: Primary health care centres
PPI: Proton pump inhibitors
SD: Standard deviation
SIDIAP: Information system for research in primary care
