# Correction to: Multimorbidity patterns, polypharmacy and their association with liver and kidney abnormalities in people over 65 years of age: a longitudinal study

**DOI:** 10.1186/s12877-021-02567-2

**Published:** 2022-05-06

**Authors:** Noemí Villén, Marina Guisado-Clavero, Sergio Fernández-Bertolín, Amelia Troncoso-Mariño, Quintí Foguet-Boreu, Ester Amado, Mariona Pons-Vigués, Albert Roso-Llorach, Concepción Violán

**Affiliations:** 1 Àrea del Medicament i Servei de Farmàcia, Gerencia Territorial de Barcelona, Institut Català de la Salut, Barcelona, Spain; 2 Methodology of Biomedical Research and Public Health, Department of Pediatrics, Obstetrics and Gynecology, and Preventive Medicine and Public Health, Universitat Autònoma de Barcelona, Barcelona, Spain; 3 Fundació Institut Universitari per a la recerca a l’Atenció Primària de Salut Jordi Gol i Gurina (IDIAPJGol), Barcelona, Spain; 4 Universitat Autònoma de Barcelona, Cerdanyola del Vallès, Bellaterra, Spain; 5 Department of Psychiatry, Vic University Hospital, Vic, Spain; 6 Àrea de Serveis Assistencials, Servei Català de la Salut, Barcelona, Spain

**Correction to: BMC Geriatr 20, 206 (2020)**

**https://doi.org/10.1186/s12877-020-01580-1**

In the original publication [1], the following affiliation was missing for Noemí Villén:

- Methodology of Biomedical Research and Public Health, Department of Pediatrics, Obstetrics and Gynecology, and Preventive Medicine and Public Health, Universitat Autònoma de Barcelona, Barcelona, Spain

The original article has been updated.
